# A Novel Phytochemical, DIM, Inhibits Proliferation, Migration, Invasion and TNF-α Induced Inflammatory Cytokine Production of Synovial Fibroblasts From Rheumatoid Arthritis Patients by Targeting MAPK and AKT/mTOR Signal Pathway

**DOI:** 10.3389/fimmu.2019.01620

**Published:** 2019-07-23

**Authors:** Hongyan Du, Xi Zhang, Yongchang Zeng, Xiaoming Huang, Hao Chen, Suihai Wang, Jing Wu, Qiang Li, Wei Zhu, Hongwei Li, Tiancai Liu, Qinghong Yu, Yingsong Wu, Ligang Jie

**Affiliations:** ^1^School of Laboratory Medicine and Biotechnology, Southern Medical University, Guangzhou, China; ^2^Department of Rheumatology and Clinical Immunology, Zhujiang Hospital, Southern Medical University, Guangzhou, China; ^3^Department of Toxicology, Guangzhou Center for Disease Control and Prevention, Guangzhou, China

**Keywords:** 3′3-Diindolylmethane(DIM), suppress, rheumatoid arthritis fibroblast-like synoviocytes (RA-FLSs), adjuvant-induced arthritis (AIA), MAPK, AKT/mTOR

## Abstract

In rheumatoid arthritis(RA) pathogenesis, activated RA fibroblast-like synoviocytes (RA-FLSs) exhibit similar proliferative features as tumor cells and subsequent erosion to cartilage will eventually lead to joint destruction. Therefore, it is imperative to search for compounds, which can effectively inhibit the abnormal activation of RA-FLSs, and retard RA progression.3′3-Diindolylmethane (DIM), the major product of the acid-catalyzed oligomerization of indole-3-carbinol from cruciferous vegetables, has been reported to be functionally relevant to inhibition of migration, invasion and carcinogenesis in some solid tumors. In this study, we explored the anti-proliferation, anti-metastasis and anti-inflammation effects of DIM on RA-FLSs as well as the underlying molecular mechanisms. To do this, primary RA-FLSs were isolated from RA patients and an animal model. Cell proliferation, migration and invasion were measured using CCK-8, scratch, and Transwell assays, respectively. The effects of DIM on Matrix metalloproteinases (MMPs) and some inflammatory factors mRNA and key molecules such as some inflammatory factors and those involved in aberrantly-activated signaling pathway in response to tumor necrosis factor α(TNF-α), a typical characteristic mediator in RA-FLS, were quantitatively measured by real-time PCR and western blotting. Moreover, the effect of DIM on adjuvant induced arthritis(AIA) models was evaluated with C57BL/6 mice *in vivo*. The results showed that DIM inhibited proliferation, migration and invasion of RA-FLS *in vitro*. Meanwhile, DIM dramatically suppressed TNF-α–induced increases in the mRNA levels of *MMP-2, MMP-3, MMP-8*, and *MMP-9*; as well as the proinflammatory factors *IL-6, IL-8*, and *IL-1*β. Mechanistic studies revealed that DIM is able to suppress phosphorylated activation not only of p38, JNK in MAPK pathway but of AKT, mTOR and downstream molecules in the AKT/mTOR pathway. Moreover, DIM treatment decreased expression levels of proinflammatory cytokines in the serum and alleviated arthritis severity in the knee joints of AIA mice. Taken together, our findings demonstrate that DIM could inhibit proliferation, migration and invasion of RA-FLSs and reduce proinflammatory factors induced by TNF-α *in vitro* by blocking MAPK and AKT/mTOR pathway and prevent inflammation and knee joint destruction *in vivo*, which suggests that DIM might have therapeutic potential for RA.

## Introduction

Rheumatoid arthritis (RA) is one of the prevalent systemic, inflammatory, and autoimmune diseases characterized by persistent synovitis in limb joints and resulted in bone erosion, even malformation and disability. According to reports, the disability rate from RA patients is up to 60% within 5–10 years and to 90% within 30 years. The 5-years survival rate of patients with external articular phenotype is only 50% (1). Because of the complicated immune mechanism, the RA etiology is still unclear. However, it is well-known that the terminal target of RA is synovium which characterized by Synovial hyperplasia, synovium pannus invasion and finally destroying the bone and cartilage. In RA pathogenesis activated RA-FLSs exhibit similar aggressive characteristic as tumor cells, which is the main trigger for abnormal hyperplasia and joint destruction (2, 3). Therefore, it is of great scientific significance to find pathways and targets to inhibit the proliferation and invasion of RA-FLS.

**Figure 1:**
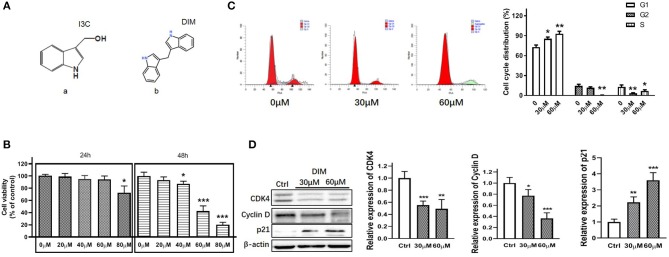
DIM suppresses the viability of RA-FLSs and arrests the cell cycle in G1 phase. **(A)** Molecular structure of (a) indole-3-carbinol (I3C) and (b) 3,3′-diindolylmethane (DIM). **(B)** The cell viability of RA-FLSs treated with DIM at various concentrations (0,20,40,60,80 μM) for 24 h and 48 h. **(C)** The cell cycle of RA-FLSs treated with DIM at various concentrations (0,30,60 μM) for 24 h. **(D)** Expression levels of CDK4, Cyclin D and p21 in RA-FLSs after treated with DIM (0,30,60 μM) for 24 h measured by Western blot(left panel). Representative images of immune blot and densitometric quantification of CDK4, Cyclin D and p21 expression(right panel). Data are represented as the mean ± SD (*n* = 3). ^*^P < 0.05, ^**^P < 0.01, ^***^P < 0.001 vs. 0 μM (Ctrl).

Many natural compounds from fruits and vegetables have been reported pharmaceutically effective against tumors and some inflammatory diseases (4, 5). Indolyl-3-carbinol (I3C) is a bioactive glucobrassicin with indole group originally found in cruciferous vegetables (Figure 1Aa). Because of its high instability, I3C is usually converted to more than 15 oligomeric and dimer bioactive compounds under acid conditions *in vivo*. Especially, 3′3-Diindolylmethane (DIM), a indole derivative (Figure 1Ab), is the unique dimer form converted from I3C and exert main function for I3C *in vivo* (6–8). Recently more and more concerns have been put on DIM due to its anti-proliferation, anti-cancer activity and anti-inflammatory effects in various cancers including oral, prostate, breast, colorectal, pancreatic, liver, and gastric cancer (9–13). Moreover, some researchers even give recommendations for DIM intake contributing to a greater understanding of exposure estimates (14).

Recurrent and chronic inflammation is involved in the development of a variety of human cancer and autoimmune diseases. Inflammation promotes the proliferation and inhibits apoptosis of related cells. It also encourages angiogenesis and production of proinflammatory cytokines in RA (1, 15). Considering that RA is a typical chronic inflammatory autoimmune disease and DIM can exert anti-inflammatory and chemopreventive effects, we muse boldly that DIM would be a potential natural product contributing for RA treatment. However, the effects of DIM on RA is still not clear. Therefore, in this study, we not only investigated the effects of DIM in treating RA in terms of proliferation, migration, invasion and producing proinflammatory cytokines but explored the underlying molecular mechanisms. Furthermore, we tried to test the effects of DIM on controlling inflammatory progress in adjuvant-induced arthritis (AIA) models.

## Materials and Methods

### Isolation and Culture of Cells

Six synovial tissues were obtained from active RA patients who were undergoing synovectomy with arthroscopy. The RA patients included 2 men and 4 women(average age 52.8 ± 14.3 years; range 24–61 years, whose detailed information were shown in Table S1. All selected RA patients fulfilled the American College of Rheumatology revised criteria of the diagnosis of RA (16) and were informed consent. This study complied with the rules enacted by the Medical Ethics Committee of the Zhujiang Hospital, Southern Medical University and was performed according to the recommendations of the Declaration of Helsinki. The harvested synovial tissue were cut into small parts and incubated with collagenase I for 1–3 h at 37°C to isolate synoviocytes (RA-FLSs) according to previous published research (17–19). RA-FLSs were cultured in DMEM/F12 supplemented with 10% fetal bovine serum (FBS), 100 U/mL penicillin, and 100 mg/L streptomycin at 37°C and 5% CO_2_. Until ~95% confluency, cells were subsequently digested using 0.25% trypsin, collected, re-suspended, and planted for proliferation. RA-FLSs obtained from passage three to six were used for the following experiments. All cell culture reagents were from Gibco^®^ (Thermo Fisher Scientific, MA, USA).

### Cell Viability Assay

The Cell Counting Kit (CCK-8) assay (KeyGEN BioTECH) was utilized to determine the cell viability according to the manufacture's instruction. RA-FLSs were planted into a 96-well plate with a density of 2.0 ×10^3^/well and cultured in 100 μL DMEM medium with 10% (v/v) FBS. Twelve hours later cells were treated with DIM(C17H14N2, ≥98% HPLC, CAS:1968-05-4, Sigma-Aldrich) at various concentration. DIM was dissolved in dimethyl sulfoxide (DMSO) and the solution was diluted to the final concentration in DMEM supplemented with 10% FBS. Cells in the control group were treated with vehicle (DMSO in DMEM supplemented with 10% FBS). After 24 and 48 h pretreated with DIM, the RA-FLSs incubated with 10 μL CCK-8 at 37°C for 2 h. The absorbance was measured at 450 nm with a microplate reader.

### Cell Cycle Analysis

The Cell Cycle Detection Kit (KeyGEN BioTECH) was utilized to determine the cell cycle according to the manufacture's instruction. Before treating RA-FLSs were serum starved for 24 h, then incubated with DIM in various concentrations for 24 h. Cells were harvested and fixed at 4°C with 70% cold ethanol overnight. Next, fixed cells were subsequently washed with PBS again and incubated with 100 μL RNase A at 37°C for 30 min. To stain the cells nuclei propidium iodide was added into suspension and incubated with cells at room temperature in a dark place for 30 min. Finally, stained cells were detected with BD FACSCalibur™ Flow Cytometer(BD Bioscience, USA) as described previously (18).

### Cell Migration and Invasion

The Boyden chamber method was used to RA-FLSs migration assay in 24-well plate with 6.5 mm diameter inserts containing 8 μm pores (Costar, New York, NY, USA). Briefly, RA-FLSs were pretreated with 1% (v/v) dimethyl sulfoxide (DMSO) and DIM respectively for 24 h. Then, cells were trypsinized and re-suspended with serum-free DMEM medium at a final concentration of 2 ×10^4^ /mL. Two hundred microliter cell suspension was put into the upper chamber of the Transwell insert. 500 μL DMED with 10% FBS as chemoattractant was placed in the lower wells. The plate was incubated at 37°C under 5% CO_2_ for 6–12 h. The non-migrating cells remaining on the upper surface of the chamber were removed using a cotton swab. The cells adhering beneath the chamber, which went through the filter, were fixed with methanol for 15 min and stained with 0.1% crystal violet for 15 min. The filter was removed carefully with a knife and fixed on a glass slide with resin. The cells was quantified by counting the stained cells that migrated to the lower side of the filter using an optical microscope. The stained cells were counted as the mean number of cells per 6 random fields for each assay.

For the *in vitro* invasion assay, similar experiment was performed using inserts coated with Matrigel basement membrane matrix (BD Biosciences, Oxford, UK). Finally, the stained cells were counted as the mean number of cells per 6 random fields for each assay. All of the experiments were replicated 3 times.

### Wound Healing Assay

The first day RA-FLSs were planted into a 12-well culture plate and grown to confluence up to more than 90%. Next day the culture media were replaced with fresh DMEM within various concentration DIM individually. The plate was then scratched with a sterile plastic pipette tip and washed with PBS twice to remove deciduous cells. There was a single wound was created in the center of the cell monolayer. After 48 h the wound areas were respectively photographed using microscope (Olympus IX51, Japan) equipped with a digital camera, and three assays of wound area were made at randomly fields. The extent of wound closure was presented as the percentage by which the original scratch area had decreased at each measured time point. The data are presented as the mean ± SD of three independent experiments.

### RNA Isolation and Real-Time PCR Analysis

To measure the effect of DIM on RA-FLSs some cytokines and MMPs expression were detected by real-time PCR analysis as described previously (20). Cells were seeded in 12-well plates at a density of 5 ×10^4^/well for overnight and treated with TNF-α (10 ng/mL) or/and DIM(25 and 50 μM) for 24 h. Total RNA was isolated using Trizol reagent (Invitrogen, San Diego, CA, USA) and cDNAs were reversely transcripted using the Prime Script RT Reagent kit (Takara Biotechnology, Dalian, China) according to the manufacturer's protocol. Quantification of expressions of human cytokines and MMPs mRNAs was determined using SYBR Premix Ex TaqTM kit (Takara Biotechnology, Dalian, China) on an ABI-7500 Thermal Cycler (Applied Biosystems Inc., Foster City, CA, USA) according to the manufacturer's instructions. All experiments were performed in triplicate and replicated 3 times and negative (ddH_2_O containing no template) controls were included. The primers for real-time PCR were listed in Table S2. To quantify the relative expression of each gene, Ct values were normalized to the endogenous β-actin (ΔCt = Ct_target_−Ct_β*-actin*_) and compared with a calibrator using the ΔΔCt method (ΔΔCt = ΔCt_sample_−ΔCt_control_).

### Western Blot Assay

The levels of protein expression were detected by western blot analysis as described previously (18). RA-FLSs were treated with 40 and 80 μM DIM for 24 h. Briefly, total cellular protein was extracted using RIPA lysis buffer and phosphatase inhibitors(Beyotime Biotechnolgoy, Nantong, China) on ice. After aspirated and thawed repeatly the lysates were separated by centrifugation at 12,000 rpm for 20 min at 4°C. The supernatants were transferred and the debris were discarded. The concentration in supernatants were detected with Pierce^®^ BCA Protein Assay Kit(Thermo Scientific, USA). Equal amounts of protein lysate were separated by 10% SDS-PAGE and then transferred to PVDF membranes. The membranes were blocked with 5% non-fat dry milk at room temperature for 1–2 h and incubated overnight at 4°C with primary antibodies. Subsequently membranes were incubated for 1 h at room temperature with secondary antibodies. Endogenous β-actin or GAPDH were used as an internal standard for normalization. The protein bands were exposed with Clarity™ Western ECL Substrate kit (Bio-Rad Laboratories, Shanghai, China) and measured by ChemiDoc^®^ XRS+ System (Bio-Rad Laboratories, Shanghai, China). The band density was quantified by Image J software. Primary antibodies included p38 MAPK, JNK, p44/42 MAPK (Erk1/2),FAK, AKT, mTOR, p70S6K, 4E-BP1, and their corresponding phosphorylation antibody, Phospho-p38 MAPK(Thr180/Tyr182), Phospho-JNK (Thr183/Tyr185), Phospho-p44/42 MAPK (Erk1/2) (Thr202/Tyr204), phospo-FAK (Tyr397), Phospho-Akt (Ser473), Phospho-p70 S6 Kinase (Thr389), and Phospho-4E-BP1 (Ser65), which were all purchased from Cell Signaling Technology, USA. Anti-β-actin and GAPDH antibodies were purchased from bioworld technology Inc.

### Measurements of Cytokines Level by ELISA

Cytokines level were measured by human enzyme-linked immunosorbent assay (ELISA) kits (Jiangsu Meimian Industrial Co., Ltd, Jiangsu, China) according to the manufacturer's instructions. For IL-6 releasing from RA-FLSs, cells were seeded into 6-well plates at a density of 5 ×10^5^/well overnight. Following treated with TNF-α (10 ng/mL) or/and DIM(25 and 50 μM) for 48 h the supernatant was collected from culture, then centrifuged (2,000 g for 10 min) and analyzed for the secretion of IL-6 using Microplate spectrophotometer(Biorad, USA) at 450 nm as described previously (20). Other Cytokines assays were performed just like IL-6 protocol. All experiments were performed in triplicate and replicated 3 times.

### Animals

Male C57BL/6 mice aged 10–12weeks were purchased from Experimental Animal Center of the Southern Medical University. Mice were fed in well ventilated cages with free access to commercial diet and tap water. The room housed mice was fixed temperature (22 ± 2) °C and humidity (50 ± 20%) under standard laboratory conditions of 12 h/12 h light/dark cycles. All the experimental procedures abided by the guidelines of ethical regulations for institutional animal care and use in the Southern Medical University and approved by The Southern Medical University Ethics Committee for Animal Laboratory Research.

### Animal Treatments

Eighteen male C57BL/6 mice (about 20 g/body weigh) were randomly divided into three groups of six, which were the normal group, the AIA model group and AIA model treated with DIM group. The induction procedures of AIA mice were refer to previously described (21–24) and adjusted in some points. The detail procedures as follows: same volume of 5% bovine serum albumin (BSA, Sigma, USA) dissolved in PBS and Freund's complete adjuvant (Sigma-Aldrich, USA) supplemented with 1.0 mg/ml heat-killed Mycobacterium tuberculosis(strain H37Ra, ATCC25177) were mixed and emulsified. On day 0, except for the normal group, mice were immunized by subcutaneously injecting 100 μL emulgator into the each side of forelimb respectively. On day 14, mice were immunized by injecting 10 μL emulgator emulsified with 5%BSA and Freund's incomplete adjuvant (Sigma-Aldrich, USA) into each sides of knee articular cavity. From day 2 to day 31 after immunizing, mice were administered with oral gavage of 100 μL DIM (10 mg/kg) suspended in 1% sodium carboxylmethyl cellulose(viscocity:600–1,000 mpa.s, USP, Shanghai Macklin Biochemical Co., Ltd.) once a day consecutively. Normal and AIA model groups were given an equal volume of 1% sodium carboxylmethyl cellulose suspension i.g. simultaneously. The mice were then monitored every day by examiners who were blinded to the experimental design. Body weight and the mediolateral knee joint diameter were measured with an electronic scale and an vernier caliper as general physical signs every 5 day (24, 25).

### Measurement of Serum Pro-inflammatory Cytokines Concentration

On day 40 and 80 after immunization, about 0.5 mL each blood samples were collected from the eyeballs of mice and allowed to clot for 1 h at room temperature. Serum was separated by centrifuging at 2,000 g for 10 min and stored at −80°C for analysis. The levels of inflammatory cytokines in serum *in vivo* were respectively detected with Mice IL-6, IL-17, TNF-α, IL-8, and IL-1β ELISA Kits (Jiangsu Meimian Industrial Co., Ltd, Jiangsu, China) according to the manufacturer's instructions, and absorbance was measured at 450 nm (26, 27).

### Spleen and Liver Indices Assays

On day 80 after immunization following blood collection, the mice anesthetized with CO_2_ were sacrificed by cervical dislocation. The spleen and liver were removed and weighed. The spleen and liver indices were expressed as the ratio of spleen and liver wet weight to mice body weight (g/g), respectively. That is, organ index = organ wet weight (g)/animal body weight (g) ×100% (27, 28).

### Histopathological Examination of Joints

The two hind limbs with knee articular of each mice were immediately cut off after removing spleen and liver. After removing the muscle tissue the joint parts were fixed in Roles-Bio^®^ Universal Tissue Fixative (Roles-Bio, Guangzhou Routh Biotechnology Co., Ltd.) for 2 days, then decalcified with Roles-Bio^®^ Quick Decalcifying Solution (Roles-Bio, Guangzhou Routh Biotechnology Co., Ltd.) for 1-2 day at room temperature. After decalcification, the tissues were dehydrated, processed, and then embedded in paraffin. Serial paraffin sections (5 μm) were stained with hematoxylin and eosin (H&E) (27) and synovitis and joint destruction were graded in a blinded manner. A histologic scoring system was used, where 1 = mild, 2 = moderate, and 3 = severe (24).

### Statistical Analysis

Results of multiple experiments were presented as the mean ± standard deviation (SD). Data analysis was performed using GraphPad 6.0 Software (GraphPad, San Diego, CA, USA). The statistical comparisons (*P*-values) between two groups were calculated using Student's *t*-test and *P*-values between more than three groups were calculated using one-way and/or two-way analysis of variance (ANOVA). *P-*values <0.05 were considered statistically significant. Number of replicates and/or total number of animals were shown in figure legends or within the figures.

## Results

### DIM Suppresses the Viability of RA-FLSs and Arrests the Cell Cycle in G1 Phase

To explore the effect of DIM on the viability of FLSs, we measured the effect of DIM with serial concentrations(0, 20, 40, 60, and 80 μM) on the viability of RA-FLSs. DIM almost did not affect cell viability except for concentrations of 80 μM after 24 h treatment, while higher concentrations DIM(40, 60, and 80 μM) showed a dose-dependent decrease in cell viability after 48 h treatment (Figure 1B). Similarly, cell cycle analysis also indicated that DIM resulted in a significant increase in proportion of cells in the G1 phase and a significant decrease in the S and G2/M phase (Figure 1C), which suggested DIM could arrest the cell cycle in G1 phase. Next, how DIM affected cell cycle regulators was explored. As shown in Figure 1D, the G1/S CDKs (CDK4) and the G1/S cyclin (cyclin D1) were all downregulated. On the contrary, p21, a cyclin-depend kinase inhibitor, was upregulated in RA-FLSs treated with DIM. Collectively, these results suggest that DIM could suppress the proliferation of RA-FLSs and arrest the cell cycle in G1 phase through regulating cell-cycle proteins.

### DIM Suppresses the Migration and Invasion of RA-FLSs

**Figure 2:**
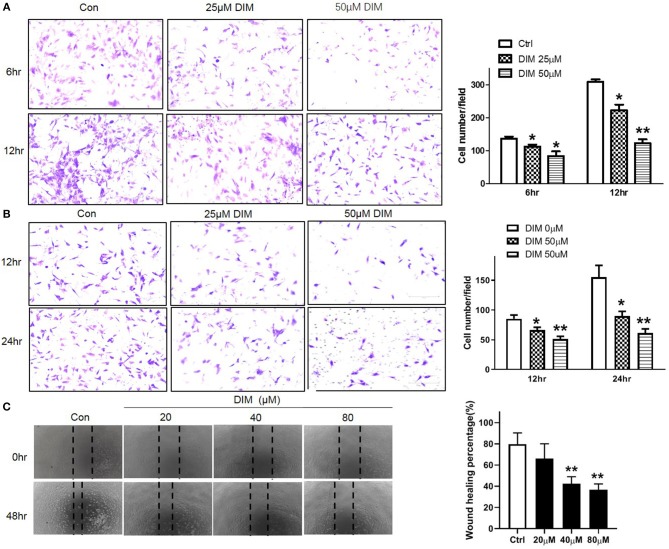
DIM Suppresses the migration and invasion of RA-FLSs. **(A)** The effect of DIM (0, 25, 50 μM) on migration was detected with transwell Boyden chamber after 6 and 12 h. The images are representative of migration or invasion through the membrane after staining. Original magnification 200 × (left panel). Cell numbers/field are presented as the mean ± SD of six independent fields (right panel). **(B)** The effect of DIM (0, 25, 50 μM) on invasion was detected with transwell Boyden chamber coated with a Matrigel basement membrane matrix after 12 and 24 h. The images are representative of migration or invasion through the membrane after staining. Original magnification 200 × (left panel). Cell numbers/field are presented as the mean ± SD of six independent fields (right panel). **(C)** The effect of DIM (0, 20, 40, 80 μM) on wound healing was detected with cell scratching assay. After 48 h the wound area was photographed using microscope. Original magnification 100 × (left panel). The extent of wound closure was presented as the percentage by which the original scratch width had decreased at each measured time point (right panel). The values are the mean ± SEM from at least 3 independent experiments. ^*^P < 0.05, ^**^P < 0.01 vs. 0 μM (Ctrl).

To evaluate the effect of DIM on migration and invasion *in vitro*, the migration and invasion of RA-FLSs were evaluated using the transwell Boyden chamber and wound closure assays. 25 and 50 μM DIM treatment markedly decreased both migratory and invasion capacity of RA-FLSs comparing with control as shown in Figures 2A,B. This result was further confirmed by wound healing assay. Although 20 μM DIM did not influence the ability of RA-FLSs migrating from one end of wound to the other, higher concentration DIM (40 and 80 μM) did reduce the wound healing ability significantly as shown in Figure 2C. All of which indicated DIM could suppress the migration and invasion of RA-FLSs *in vitro*.

### DIM Suppresses the Pro-inflammatory Cytokines and MMPs Expression

**Figure 3:**
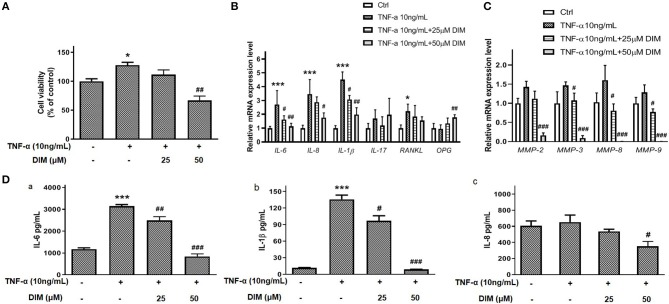
The effect of DIM on producing pro-inflammatory cytokines and MMPs of RA-FLSs. **(A)** The effect of DIM (25 and 50 μM) on cell viability induced by TNF-α(10 ng/mL). **(B)** The effect of 25 and 50 μM DIM on relative mRNA expression of *IL-6, IL-8, IL-1*β*, IL-17, RANKL*, and *OPG* induced by TNF-α (10 ng/mL) to β-actin in RA-FLSs. **(C)** The effect of DIM (25and 50 μM) on relative mRNA expression of *MMP-2, MMP-3, MMP-8*, and *MMP-9* induced by TNF-α (10 ng/mL) to β-actin in RA-FLSs. **(D)** The effect of DIM (25 and 50 μM) on IL-6, IL-8, and IL-1β releasing in culture supernatant induced by TNF-α(10 ng/mL) in RA-FLSs. (a) The effect of DIM (25 and 50 μM) on IL-6 releasing induced by TNF-α (10 ng/mL) determined by ELISA. (b) The effect of DIM (25 and 50 μM) on IL-1β releasing induced by TNF-α (10 ng/mL) determined by ELISA. (c) The effect of DIM (25 and 50 μM) on IL-8 releasing induced by TNF-α (10 ng/mL) determined by ELISA. The values are the mean ± SEM from at least 3 independent experiments. ^*^P < 0.05, ^***^P < 0.001 vs. Ctrl(0 μM DIM and 0 ng/mL TNF-α). ^#^*P* < 0.05, ^*##*^*P* < 0.01, ^*###*^*P* < 0.001 vs. group treated by TNF-α(10 ng/mL).

The pro-inflammatory cytokines and matrix metalloproteinases(MMPs) play important roles on proliferation, migration and invasion of RA-FLSs and even erosion of cartilago articularis (15). TNF-α is a key pro-inflammatory cytokine contributing to RA-FLSs surviving and developing arthritis. In our results shown in Figure 3A, TNF-α promoted the viability of RA-FLSs and although 25 μM DIM did not decrease the cell viability induced by TNF-α(10 ng/mL), 50 μM DIM did. To determine the role of DIM on main pro-inflammatory cytokines expression, the mRNA levels of *IL-6, IL-8, IL-1*β*, IL-17, Receptor Activator of Nuclear Factor-*κ *B Ligand (RANKL)* and special *Osteoprotegerin(OPG)* stimulating by TNF-α in RA-FLSs were measured with quantitative PCR after treated with 25 and 50 μM DIM for 24 h. As shown in Figure 3B, except for *OPG* the mRNA expression of *IL-6, IL-8, IL-1*β, and *RANKL* in RA-FLSs up-regulated more or less after induced by TNF-α(10 ng/mL), but DIM(25 and 50 μM) inhibited *IL-6, IL-8*, and *IL-1*β mRNA expression levels increase caused by TNF-α(10 ng/mL) and had no significant effect to *RANKL*. Neither TNF-α nor DIM treatment obviously altered *IL-17* mRNA expression. Distinguishingly, 50 μM DIM promoted the mRNA expression of *OPG* comparing to control and TNF-α treatment groups. As shown in Figure 3C, the mRNA expression of *MMP-2, MMP-3, MMP-8*, and *MMP-9* were very less and almost undetectable after DIM(25 and 50 μM) treatment, which indicated DIM profoundly inhibited increase in mRNA expression of *MMP-2, MMP-3, MMP-8*, and *MMP-9* induced by TNF-α in RA-FLSs.

Furthermore, Except for mRNA level the effect of DIM on some pro-inflammatory cytokines release induced by TNF-α was also explored. After treating with 25 and 50 μM DIM for 48 h, cells culture supernatant were collected and ELISA assays for IL-6, IL-8, IL-1β, and IL-17 were performed. The data in Figure 3D indicated TNF-α(10 ng/mL) significantly up-regulated the IL-6 and IL-1β release from RA-FLSs especially IL-6, but DIM treatment could inhibit their increase in culture supernatant (Figures 3Da,b). Interestingly, TNF-α(10 ng/mL) stimulation did not cause profound increase in IL-8 releasing, 50 μM DIM indeed down-regulated IL-8 expression (Figure 3Dc). IL-17 was undetectable in this ELISA-based assay. Collectively, these results suggest that DIM may contribute to reduce producing and releasing some pro-inflammatory cytokines and MMPs in RA-FLSs.

### DIM Suppresses the Activation of MAPK and Akt/mTOR Pathways Induced by TNF-α in RA-FLSs

**Figure 4:**
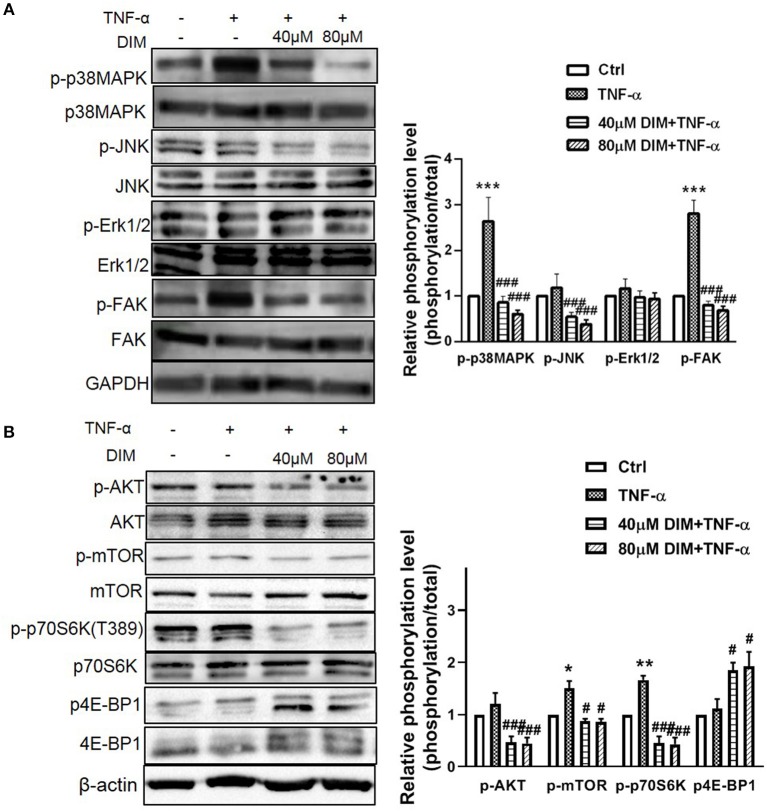
The effect of DIM on the intracellular phosphorylated activation of MAPK and Akt/mTOR pathway induced by TNF-α in RA-FLSs. **(A)** RA-FLSs were treated with TNF-α(10 ng/mL) or/and DIM (40 and 80 μM) for 24 h, western blot analysis was conducted to assess the expression and phosphorylation level of p38MAPK, JNK, ERK, and FAK. Representative images of immune blot (left panel) and densitometric quantification phosphorylation/total of p38MAPK, JNK, ERK, and FAK expression(right panel). **(B)** RA-FLSs were treated with TNF-α(10 ng/mL) or/and DIM (40 and 80 μM) for 24 h, western blot analysis was conducted to assess the expression and phosphorylation level of AKT, mTOR, p70S6K and 4E-BP1. Representative images of immune blot (left panel) and densitometric quantification phosphorylation/total of AKT, mTOR, p70S6K and 4E-BP1 expression(right panel). Densitometry analysis from three independent experiments was used to quantitate the protein expression. ^*^P < 0.05, ^**^P < 0.01, ^***^P < 0.001 vs. Ctrl (0 μM DIM), ^#^*P* < 0.05, ^*##*^*P* < 0.01, ^*###*^*P* < 0.001 vs. group treated by TNF-α(10 ng/mL).

Mitogen Activated Protein Kinases (MAPK), including ERK, JNK, and P38MAP kinase (P38), play the major role in stress-induced cellular responses such as cell proliferation, survival, apoptosis and invasion and are intracellular effector molecules that are embedded in a highly active signaling cascade in RA-FLSs (29). Since DIM could suppress viability and proliferation of RA-FLSs, we presume that MAPK pathway was affected by DIM in RA-FLSs. Therefore, the effect of DIM on MAPKwas investigated. The cells were treated with 10 ng/mL TNF-α in the presence or absence of 40 and 80 μM DIM for 24 h, western blot analysis was conducted to assess the expression and phosphorylated levels of p38MAPK, JNK, ERK. According to results in Figure 4A the DIM could obviously decrease the phosphorylation level of p38MAPK, JNK induced by TNF-α, however, had no effect on ERK phosphorylated activation. Therefore, detailed investigations of components of MAPK have shown that DIM strongly reduced p38MAPK, JNK activity, which may be responsible for controlling abnormal hyperplasia caused with RA-FLSs. There are some evidences from studies activation of the FAK family signaling cascade in RA lining cells contributed to cell adhesion and migration into the diseased synovial tissue (30). Therefore, how DIM regulated FAK signaling was studied. As shown in Figure 4A, the levels of non-phospho FAK were not changed in all groups after stimulating with TNF-α but that of phospho-FAK was decreased in RA-FLSs treated with DIM.

In addition, AKT/mTOR pathway in promoting aggressive immune-cells and synoviocytes proliferation and survival plays an important role in progress of RA (31). To clear the effect of DIM on the Akt/mTOR pathway in RA-FLSs, the phosphorylation level of Akt, mTOR and downsteam p70S6K and 4E-BP1 were determined. Although the Akt phosphorylated activation was not obviously increased after treated with TNF-α, DIM indeed decreased the phosphorylation of Akt and mTOR (Figure 4B). Moreover, DIM also respectively, down-regulated and up-regulated the phosphorylated level of downstream p70S6K and 4E-BP1 stimulating with TNF-α (Figure 4B), which suggested DIM suppressed the Akt/mTOR pathway for cell growth and proliferation. Altogether, DIM affected the biological behaviors of RA-FLSs via suppressing intracellular phosphorylated activation of MAPK and Akt/mTOR pathway.

### DIM Ameliorates Arthritis Severity in Mice With AIA

#### The Effect of DIM on the Weight and Knee Joint Diameter of the Mice

**Figure 5:**
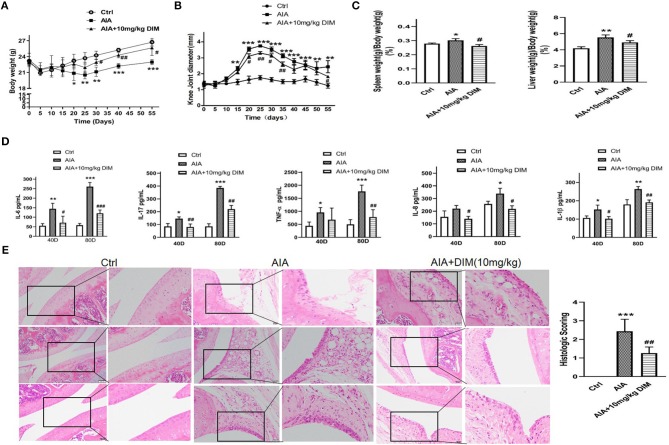
DIM ameliorates arthritis severity in mice with AIA. **(A)** The effect of DIM on mean change in body weight of mice after immunization (from day 0 to day 55) monitored every 5 days. **(B)** The effect of DIM on mean change in knee joint diameter after immunization (from day 0 to day 55) monitored every 5 days. **(C)** The effect of DIM on the spleen and liver index of mice with AIA and control. Data shown as spleen or liver weight (g)/body weight (g) ×100%. **(D)** The effect of DIM on IL-6, IL-17, TNF-α, IL-8, and IL-1β expression in serum of mice with AIA and control after immunization on day 40 and 80. **(E)** The effect of DIM on the pathohistological features of knee joints in mice with AIA. Photomicrographs for knee joint sections stained with H&E (left panel, original magnification 400× ). The scores for inflammatory severity (right panel). All the data were expressed as means ± S.D. *n* = 6, ^*^P < 0.05, ^**^P < 0.01, ^***^P < 0.001 vs. Ctrl (normal mice group), ^#^*P* < 0.05, ^*##*^*P* < 0.01, ^*###*^*P* < 0.001 vs. AIA model group.

The effect of DIM on mean change in body weight of mice after immunization (from day 0 to day 55) monitored every 5 days. During the experiment the mice from three groups were eating and drinking normally. As shown in Figure 5A, on the day 20 after immunization the body weight of mice with AIA obviously occurred decrease comparing to normal group. However, the treatment of the mice with DIM (10 mg/kg) via gavage had significant difference comparing with AIA group at day 30 after immunization.

Synchronously, the effect of DIM on arthritis development by measurement of the knee joint diameter was followed every 5 days. The mean diameter of knee joints from AIA group mice increased rapidly from the 15th day after immunization because of obvious swelling and at the 25th day the mean diameter was up to peak value. After that the mean diameter gradually declined. However, the mice from DIM treatment group decreased the mean diameter of knee joints comparing with mice form AIA group from the 20th day (Figure 5B).

#### The Effects of DIM on Spleen and Liver Indices in AIA Mice

To evaluate the DIM effect on immune organs the spleen and liver indices in mice were calculated as shown in Figure 5C. Compared with the normal group, the spleen and liver indices of AIA model group mice significantly increased. However, compared with the AIA model group, DIM (10 mg/kg) significantly reduced spleen and liver indices in AIA mice.

#### The Effect of DIM on Pro-inflammatory Cytokines Expression in AIA

To explore how the DIM affecting the proinflammatory cytokines in AIA, the IL-6, IL-17, TNF-α, IL-8, and IL-1β expressions in serum of AIA mice with and without DIM treatment after immunization on day 40 and 80 were detected by ELISA. The results were shown in Figure 5D. Firstly, IL-6 expression in mice from AIA model group were obviously increased comparing to ones from normal control group. Moreover, the IL-6 expression was inhibited in mice treated with DIM comparing to AIA model group both on day 40 and 80 after immunization. Next, the similar trends were observed in IL-17 IL-8 and IL-1β expressions in three groups. Although no difference in IL-8 was witnessed between normal mice and AIA mice group on day 40, there was difference occurred on day 80. The last, The data indicated that despite DIM had no notable effect on TNF-α expression on day 40, it did significantly decrease the TNF-α expression in AIA mice on day 80. Altogether, DIM (10mg/kg) could inhibit expression of the proinflammatory cytokines, IL-6, IL-17, TNF-α, IL-8, and IL-1β, in serum of AIA mice.

#### The Effect of DIM on the Pathohistological Features on Knee Joints and Arthritis Severity in AIA Mice

The mice knee joints were removed after euthanization on day 80 for histological examination of tissue sections, and then pathohistological sections were stained with H&E. As shown in Figure 5E, the knee joint from normal control group appeared clear and complete histological architecture under the microscope, whereas the knee joints from AIA mice showed abnormal histological architecture covering synovial hyperplasia, infiltration of massive inflammatory cells, together with angiogenesis (increased microvessel density) and degradation of epithelial cell. The mice from DIM (10 mg/kg i.g.) group were preserved almost normal histological architecture of the knee joints with mild synovial hyperplasia, less inflammatory cells infiltration and less erosion of synovial tissues. Meanwhile, the pathohistological score was remarkably decreased in DIM group compared with model group. All the data suggested that DIM did ameliorate arthritis severity in mice with AIA and had a potential anti-arthritic efficacy.

## Discussion

RA is a chronic autoimmune disease characterized by a hyperplastic, aggressive and invasive phenotype and involved in the formation of pannus angiogenesis, cartilage degradation, and bone erosion (32). Currently used anti-RA drugs have been found to have many side effects because they have high toxic reaction *in vivo*. It is urgent to find some natural products with less toxicity to inhibit inflammatory reaction and provide a quality of life to the patients suffering from RA.

Many fruits and vegetables contain natural bioactive components that could affect multiple signaling pathways. This feature gives these natural products pharmaceutical potential in treating some diseases where the related signaling pathways go wrong (4, 5). DIM, an indole derivative, is a bioactive compound found in cruciferous vegetables and converted from I3C in an aqueous and gastric-acidic environment (6). I3C initially drew many attentions due to its anti-cancer function. But it later was found that DIM, the converted version of I3C in body with longer half-life, was the functional ingredient exerting anti-cancer effects (7, 8). During uncovering the mechanism of DIM, anti-inflammatory effect surfaced (9). Inflammation promotes cellular proliferation, angiogenesis, inhibits apoptosis, and induces DNA damage, increasing the risk of developing disease related inflammation. DIM exhibited a significant anti-inflammatory activity was associated with reduction of pro-inflammatory cytokines (11, 33).

Due to its anti-inflammatory activity, we creatively tried to apply DIM in treating RA. According to our experiments, the DIM could in a dose-dependent manner suppress the proliferation, migration and invasion of RA-FLSs had been identified, which coincided with reports from variety of cancers research (9, 11, 34, 35). In the molecular level, DIM can arrest G1-phase cell cycle by reducing CDK4, and Cyclin D1 and elevating p21, a CDK (cyclin-dependent kinase) inhibitor.This finding is in line with previously reported mechanisms of DIM in gastric cancer and esophageal squamous carcinoma (36, 37). All of these suggest DIM suppresses cell proliferation and arrests cell cycle at G1 phase via affecting related cell cycle proteins expression. Meanwhile, some researchers found DIM could induce apoptosis of some cancer cells through both the intrinsic and extrinsic pathway by activating Caspase 8 (9, 38).

However, we did not find DIM could induce apoptosis of RA-FLSs (Figure S1). We speculated that it may be due to differences in cell types or/and individual differences.

RA-FLSs usually secret various proinflammatory cytokines and chemokines, especially IL-6, IL-8, IL-1β, TNF-α, and IL-17 to recruit and activate more immune cells to the inflammatory microenvironment, and thereby are responsible for cartilage damage and joint destruction (39, 40). TNF-α is one of the major mediators involved in RA. It had also been confirmed stimulation of 10 ng/mL TNF-α resulted in the activation of RA-FLSs and increased production of inflammatory cytokines (17, 41). In our study, we got the similar results with 10 ng/mL TNF-α. Whereas, DIM inhibited the increase both cell viability and mRNA expression and release of IL-6, IL-8, and IL-1β. Therefore, we consider DIM play the anti-inflammatory role via inhibiting pro-inflammatory cytokines at least in part. No change has been observed in *IL-17* mRNA expression upon TNF-α stimulation and DIM treatment in our RA-FLSs system. We thought it might have something to do with individual patient differences. In addition, DIM also increased the *OPG* mRNA expression in RA-FLSs, which suggested DIM was related to protection from bone damage. Nevertheless, uncovering more mechanisms has to do further work. From ELISA results we noticed although the tendency of IL-1β increase induced by TNF-α was superficially suppressed with DIM. The amount is so low that it is beyond the detection capacity of ELISA, which could account for the failure to detect IL-1β in culture supernatant.In inflammatory diseases, the degradation of synovial collagen in RA maybe related to the expression of MMPs in fibroblasts in synovial joints (42). More than fifteen synovial MMPs are reportedly expressed in RA patients, mainly including collagenase, gelatinase, and matrix metalloproteinase (43). DIM inhibited the mRNA expression of collagenase *MMP-8*, gelatinase *MMP-2* and *MMP-9*, and matrix metalloproteinase *MMP-3*. Although we could not detect these MMPs in protein level, probably due to the undetectably low amount, it indeed implied that suppression of MMPs could be the core of DIM inhibiting migration and invasion of RA-FLSs.

In addition, as for migration and invasion, FAK (focal adhesion kinase), a non-receptor tyrosine kinase, promotes cell motility, survival, and proliferation through kinase-dependent and -independent mechanisms (44). There is research on DIM treating liver cancer cells indicated DIM inhibits the migration, invasion, and metastasis of HCC cells via inhibiting phosphorylation of focal adhesion kinase (FAK, tyr397) with decreased expression of MMP-2 and MMP-9 (12), which is basically consistent with our data.

It has been confirmed DIM influences the proliferation, migration, and invasion of cancer cells through multiple signaling pathways. But what's happened in RA-FLSs caused by DIM need to be uncovered. Mitogen-activated protein kinases(MAPK) is a family of serine/threonine protein kinases widely conserved among eukaryotes and involved in many cellular key programs (45). Moreover, MAPK pathway takes part in the activation of RA-FLSs (15). We found DIM blocked the phosphorylated activation of p38 and JNK stimulated by TNF-α, but not the ERK in RA-FLSs, suggesting that the p38 and JNK pathway might mediate the action of DIM in RA-FLSs. Indeed, p38 and JNK are expressed and activated in synovial tissue from patients with RA. They not only regulates RA-FLSs growth, apoptosis and differentiation but also are critical for synovial inflammation and joint destruction in inflammatory arthritis (15, 41). There were other reports that DIM inhibited cell growth by downregulation of Akt/FoxM1 signaling pathway in gastric cancer (46), by inactivation of β-catenin/c-Myc in colorectal cancer (47). These results suggest that DIM might possess a cell type-specific function in modulating cell growth. Moreover, JNK pathway and its downstream proteins are also involved in cell migration. Additionally, FAK, a movement-associated signal pathway, finally converge at JNK, and JNK activation predicted the development of bone erosion in RA (48).

Akt is also contributed to the growth of cells via phosphorylating the downstream mTOR complex 1 (mTORC1), a translator of mRNAs to protein by means of p70S6K-S6 and 4E-BP1-eIF4E pathways (49). The mammalian target of rapamycin (mTOR) signaling pathway plays an important role in regulating a variety of cellular functions at the interface of cell metabolism, growth, and differentiation. mTOR signaling pathway is involved in the regulation of RA-FLSs invasion and might be a new target for RA therapy (50). Many investigations had demonstrated that mTOR was activated in the rheumatoid synovium especially induced by TNF-α (51). It was worth mentioning that DIM blocked the activation of AKT/mTOR and downstream p70S6K-S6 and 4E-BP1 caused by TNF-α was verified in our data, which was first direct evidence on DIM affecting AKT/mTOR pathway. This provides proofs for the anti-arthritic properties of DIM and corroborates its potential use for the treatment of RA.

In addition to the cell signaling pathway on DIM affecting there are some researches on the effect of DIM on microRNA in some cancer cells (52). MiRNA21 and miR-150-5P had been identified as target of DIM in cancer cells, which indicated that DIM could be used in target-based therapy and also as a lead for further development of potent small molecule miRNA antagonist (27, 53). Considering miRNAs play important roles in development and progression of RA, and then there are a lot of work need to discover key microRNA influenced by DIM in RA.

As a typical animal model of RA, AIA has been used in many studies to survey the pathogenesis of arthritis and to find potential therapeutic targets (25, 54). The decrease in arthritis looks like small shown in Figure 5B but the reduction in inflammatory cytokines in the DIM-treated mice is significant. We speculate that swelling of the joints is only part of the appearance of arthritis and does not indicate the severity of the overall inflammation. Moreover, the swelling of the joints is local inflammatory character and the inflammatory cytokines detected from serum are related with inflammatory of whole body. Additionally, during the peak of inflammatory response we measured the inflammatory cytokines at 28th day, but there was no significant difference between AIA model group and AIA model treated with DIM group. There was report that DIM could alleviate oxazolone-induced colitis through Th2 and Th17 suppression and Treg induction (55). Therefore, we speculate that the mechanism of DIM is not like a non-steroidal anti-inflammatory drugs, which paly direct and quick anti-inflammatory role *in vivo*. It is worth to make further study to uncover the mechanism of DIM from another angle. Moreover, it is usual that there is a lag between swelling of the joints and the inflammatory cytokines up-regulation and down-regulation, which is similar with clinical manifestations of some inflammation. Our results from the mice with AIA that DIM not only decreased the swell of knee joint caused with arthritis and down-regulated the proinflammatory factors, but improved pathohistological features, which further support our initial suppose *in vivo* that DIM may have potential to control synovial joint destruction in RA. In conclusion, our study provides a novel insight into the specific role of DIM on TNF-dependent arthritogenesis, offering *ex vivo* and *in vivo* evidence on DIM functions in regulating the proliferation, migration and invasion of RA-FLSs by inhibiting the MAPK and AKT/mTOR pathway and ameliorating arthritis severity and pathological outcome in an AIA model. Therefore, DIM may harbor a huge therapeutic and pharmaceutical potential in treating RA. Furthermore, DIM, in combination with other compounds inducing RA-FLSs apoptosis, may give a better therapeutic effect as well as improve the quality life of RA patients.

## Data Availability

The raw data supporting the conclusions of this manuscript will be made available by the authors, without undue reservation, to any qualified researcher.

## Ethics Statement

This study complied with the rules enacted by the Medical Ethics Committee of the Zhujiang Hospital, Southern Medical University and was performed according to the recommendations of the Declaration of Helsinki. All the animal experimental procedures abided by the guidelines of ethical regulations for institutional animal care and use in the Southern Medical University and approved by The Southern Medical University Ethics Committee for Animal Laboratory Research.

## Author Contributions

HD, WZ, YW, and LJ designed the research. HD, XZ, YZ, XH, JW, and QL performed the experiments. HC and SW contributed analysis and animal model construction. HD, HL and LJ analyzed the experimental data. HD, YW, and LJ wrote and revised the manuscript. All authors read and approved the final manuscript.

### Conflict of Interest Statement

The authors declare that the research was conducted in the absence of any commercial or financial relationships that could be construed as a potential conflict of interest.

## Supplementary Material

Figure S1 The effect of DIM on apoptosis of RA-FLSs. There is almost no cell apoptosis occurred after treated with 0,30,60,90 μM DIM, which indicated that DIM could not induce RA-FLSs apoptosis.

Table S1 The information of RA patients whose tissue samples were used in our research.

Table S2 The primers of human cytokines and MMPs for mRNA expression assay.
