# Supplementary figures and images for: A Novel Phytochemical, DIM, Inhibits Proliferation, Migration, Invasion and TNF-α Induced Inflammatory Cytokine Production of Synovial Fibroblasts From Rheumatoid Arthritis Patients by Targeting MAPK and AKT/mTOR Signal Pathway
Source: Front Immunol. 2019 Jul 23;10:1620. doi: 10.3389/fimmu.2019.01620 (PMC6663984; doi:10.3389/fimmu.2019.01620)

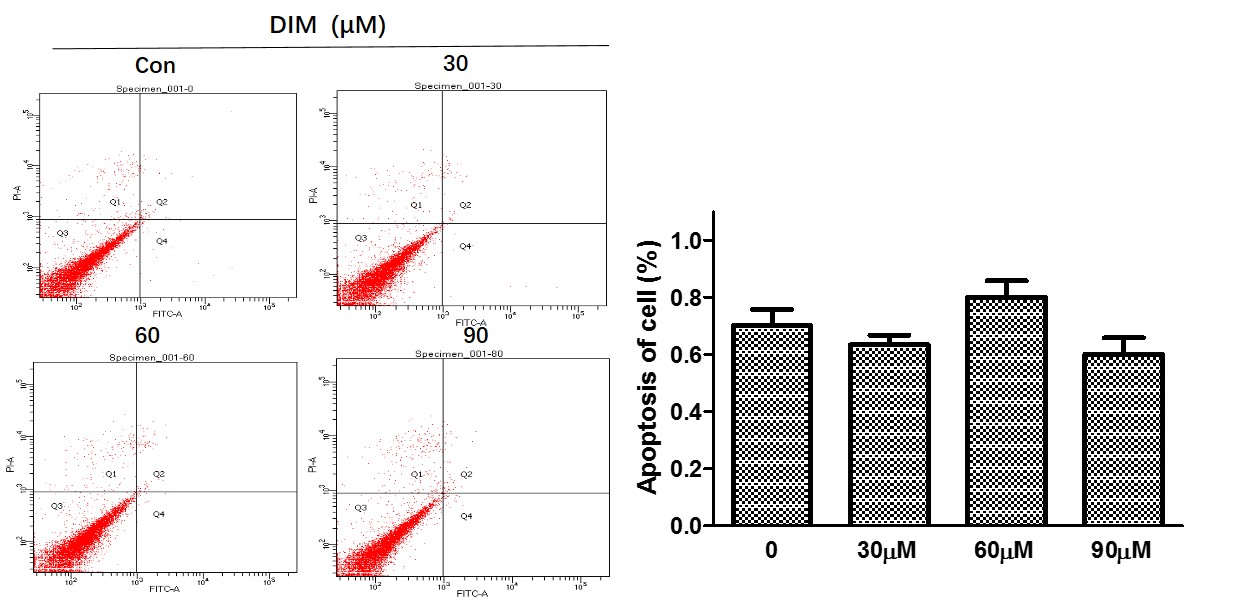

Supplement: Figure S1 — The effect of DIM on apoptosis of RA-FLSs. There is almost no cell apoptosis occurred after treated with 0,30,60,90 μM DIM, which indicated that DIM could not induce RA-FLSs apoptosis. [file Image_1.JPEG]
