# Supplementary material for: A Novel Phytochemical, DIM, Inhibits Proliferation, Migration, Invasion and TNF-α Induced Inflammatory Cytokine Production of Synovial Fibroblasts From Rheumatoid Arthritis Patients by Targeting MAPK and AKT/mTOR Signal Pathway
Source: Front Immunol. 2019 Jul 23;10:1620. doi: 10.3389/fimmu.2019.01620 (PMC6663984; doi:10.3389/fimmu.2019.01620)
Supplement: Table S1 — The information of RA patients whose tissue samples were used in our research. [file Table_1.DOCX]

**SUPPLEMENTARY MATERIAL**

**Table S1. The information of RA patients**

**whose tissue samples were used in our research**

| NO. of Patients | | Gender | | Age | | RF  (IU/mL) | | ESR | | C-reactin protein  (mg/L) | | ACPA |
| --- | --- | --- | --- | --- | --- | --- | --- | --- | --- | --- | --- | --- |
| 1 | female | | 60 | | 4760 | | 60 | | 127 | | Positive | |
| 2 | female | | 61 | | 795 | | 23 | | 109 | | Positive | |
| 3 | male | | 55 | | 948 | | 103 | | 85.3 | | Positive | |
| 4 | female | | 24 | | 678 | | 90 | | 45.9 | | Positive | |
| 5 | female | | 60 | | 202 | | 56 | | <3.14 | | Positive | |
| 6 | male | | 57 | | 998 | | 29 | | 28.6 | | Positive | |

Remark: Rheumatoid factor(RF); Erythrocyte sedimentati on rate(ESR); Anti-cyclic citrullinated peptide antibodies(ACPA)
