# Supplementary material for: A Novel Phytochemical, DIM, Inhibits Proliferation, Migration, Invasion and TNF-α Induced Inflammatory Cytokine Production of Synovial Fibroblasts From Rheumatoid Arthritis Patients by Targeting MAPK and AKT/mTOR Signal Pathway
Source: Front Immunol. 2019 Jul 23;10:1620. doi: 10.3389/fimmu.2019.01620 (PMC6663984; doi:10.3389/fimmu.2019.01620)
Supplement: Table S2 — The primers of human cytokines and MMPs for mRNA expression assay. [file Table_2.DOCX]

**SUPPLEMENTARY MATERIAL**

**Tabel S2. The primers of human cytokines and MMPs**

**for mRNA expression assay**

| Gene Name |  | Sequence | Number of bases(bps) |
| --- | --- | --- | --- |
| IL-6 | Forward | AGTGAGGAACAAGCCAGAGC | 20 |
|  | Reverse | AGCTGCGCAGAATGAGATGA | 20 |
| il-8 | Forward | AGAAGTTTTTGAAGAGGGCTGAGA | 25 |
|  | Reverse | AGTTTCACTGGCATCTTCACTGATT | 25 |
| il-1β | Forward | CCACCTCCAGGGACAGGATA | 20 |
|  | Reverse | AACACGCAGGACAGGTACAG | 20 |
| IL-17 | Forward | CTGTCCCCATCCAGCAAGAG | 20 |
|  | Reverse | AGGCCACATGGTGGACAATC | 20 |
| rankl | Forward | AACACGCGTATTTACCTGCCA | 21 |
|  | Reverse | CCATAGCCCACATGCAGTTTC | 21 |
| opg | Forward | CTACACAGACAGCTGGCACA | 20 |
|  | Reverse | ACTCCTGCTTGACGTACTGC | 20 |
| mmp-2 | Forward | TCGCCCATCATCAAGTTCCC | 20 |
|  | Reverse | GGGCAGCCATAGAAGGTGTT | 20 |
| MMP-3 | Forward | TCCGACACTCTGGAGGTGAT | 20 |
|  | Reverse | ACTTCGGGATGCCAGGAAAG | 20 |
| mmp-8 | Forward | ATGTGACGGGGAAGCCAAAT | 20 |
|  | Reverse | AAAACCACCACTGTCAGGCA | 20 |
| mmp-9 | Forward | GGACAAGCTCTTCGGCTTCT | 20 |
|  | Reverse | TCGCTGGTACAGGTCGAGTA | 20 |
